# How Social Ties Influence Consumer: Evidence from Event-Related Potentials

**DOI:** 10.1371/journal.pone.0169508

**Published:** 2017-01-12

**Authors:** Jing Luan, Zhong Yao, Yan Bai

**Affiliations:** 1 School of Economics and Management, Beihang University, Beijing, China; 2 School of Civil Engineering, Tsinghua University, Beijing, China; 3 Resource and Environment Institute of Standardization, China National Institute of Standardization, Beijing, China; Hong Kong Polytechnic University, XX

## Abstract

A considerable amount of marketing research has reported that consumers are more saliently influenced by friends (strong social ties) than by acquaintances and strangers (weak social ties). To shed light on the neural and psychological processes underlying such phenomenon, in this study we designed an amended S1-S2 paradigm (product-[reviewer-review]) that is based on realistic consumer purchase experiences. After incoming all given information (product, reviewer, review), participants were required to state their purchase intentions. The neurocognitive and emotional processes related to friend and stranger stimuli were delineated to suggest how social ties influence consumers during their shopping processes. Larger P2 (fronto-central scalp areas) and P3 (central and posterior-parietal scalp areas) components under stranger condition were elicited successfully. These findings demonstrate that the cognitive and emotional processing of friend and stranger stimuli occurs at stages of neural activity, and can be indicated by the P2 and P3 components. Electrophysiological data also support the hypothesis that different neural and emotional processing magnitude and strength underlie friend and stranger effect in the context of consumer purchase. During this process, the perception of stimuli evoked P2, subsequently emotional processing and attention modulation were activated and indicated by P2 and P3. The friend dominated phenomenon can be interpreted as the result of distinctive neurocognitive and emotional processing magnitude, which suggests that psychological and emotional factors can guide consumer decision making. This study consolidates that event related potential (ERP) methodology is likely to be a more sensitive method for investigating consumer behaviors. From the perspectives of management and marketing, our findings show that the P2 and P3 components can be employed as an indicator to probe the influential factors of consumer purchase intentions.

## Introduction

Employing event related potential technique, neuroscientific measurement to study consumers’ emotional and cognitive processes has sparked growing interest in recent years [1]. Compared with traditional empirical analysis, which may not be easy to acquire real and convictive results due to privacy and subjectivity, neurocognitive method can collect and analyze the brain’s response using precise instruments, more likely obtaining real reactions and helping further understand consumer behavior. Based on this, more interest has gradually accumulated in the consumer neuroscience research such as trustworthiness [2], consumer decision making [3,4] and emotional processing [5].

Enabled by Web 2.0 technology, the increased popularity of social networks such as Facebook, Twitter and LinkedIn, has promoted the emergence of a new platform in e-commerce called social commerce. It generally refers to the delivery of e-commerce activities and transactions via social environment mostly in social networks [6]. To summarize, it is a combination of commercial and social activities [6–8] and outstands its merits of social interactives and social influence. Compared with traditional e-commerce, where consumer reviews are shared with unknown online shoppers, in social commerce context, it focuses on sharing information with friends on social network sits [7]. Because reviews from friends are regarded as more valuable and trustworthy, it may influence online purchase and play a pivotal role in social commerce [9]. Prior marketing studies have asserted that friends (strong social ties) are more influential than acquaintances and strangers (weak social ties) in consumer decision making [9–11]. However these studies concluded from traditional empirical analysis such as self-reported survey, they cannot give further explanation about such consumer behaviors.

Thanks to the advances in brain imaging technology which allows us to enhance our knowledge about how consumers process different social tie stimuli and make decision. Particularly in social commerce context where strong social ties predominate, revealing the tie strength effect from the neuroscientific dimension will bring a novel perspective on social commerce management and practice. Therefore, this paper intends to close the gap by examining the friend dominant phenomenon from the neurocognitive level.

Employing neurocognitive methodologies such as electroencephalography (EEG) in marketing related studies can date back to the early 1970s. And with the development of neuroimaging techniques and the anatomical discovery about potential relevance of certain brain region function, an increasingly precise identification of neural responses has been enabled. As one of the most commonly used neuroimaging techniques, EEG has the outstanding advantage of high temporal resolution to detect the subtle changes in brain activity at relatively low costs. Therefore by using EEG technology many researchers have adopted tie strength (friend and stranger) into their research [12,13], especially to explore consumer purchase behavior [3,4]. Specifically Wu et al. [12] designed a dictator game to observe the recipient’s evaluation of fair/unfair behavior from friends or strangers. Leng et al. [13] detected participant’s reward feedback to friends and strangers via a gambling task. Kuan et al. [3] examined the influence of “buy” information (the number of people who have bought a deal) and “like” information (Facebook friends who like a deal) on consumers’ opinions (attitude and intention) and emotions by using friends’ portraits. Bai and Yao [4] explored the influence of social commerce review (SCR, review from friend) and e-commerce review (ECR, review from stranger) on consumer purchase intention by combining friends’ or strangers’ portraits and their reviews. All these EEG research has discovered the different influence of different social ties on human decision behaviors.

In fact, the influence of emotions on human decision making has been identified and is important to be considered when we analyze decision making behavior [14,15,16]. Empirical findings from Ultimatum Game revealed that for low offers (proposer makes an offer around 20% of the total), the responder will reject the offer at about 50% chance. This is a challenge to its standard economic solution because any amount of money is preferable to none. Sanfey et al. [16] tried to interpret this phenomenon by considering both emotional and cognitive processing, and they also suggested that the magnitude and strength of the emotional and cognitive processing might help explain results in the subsequent decision. Specifically, participants rejected unfair offers because they felt angry about an offer perceived as unfair. And these negative emotions induced by unfair treatment can lead people to make an uneconomical decision (rejecting financial gain) in order to penalize their partners. Based on their research, we reckon that the variant influence of different social ties on consumer decision making would engage neural activities involved in both emotional and cognitive processing. Previous EEG research has reported the differentiated influence of different social ties on human decision behavior, but they mainly focus on the comprehensive effect and ignore to analyze participants’ direct responses to social tie stimuli. Thus in this paper we hypothesize that processing social tie stimuli (friends and strangers) would engage both cognitive and emotional processes in the brain, and the magnitude of activation in both cognitive and emotional processing might explain the subsequent consumer decisions to accept or reject a product. And we mainly concentrate on consumers’ reaction to social tie stimuli to further investigate its influence on subsequent consumer purchase decisions.

In a social commerce context, frequent social interactions among consumers and voluntarily sharing their experiences and knowledge about products and services on the Internet [17,18] have generated tremendous useful and believable online reviews, which can further influence consumers’ choices and purchasing behaviors [19]. Considering this fact and experiments designed in previous research, in order to reproduce a realistic social commerce shopping environment, we designed a S1-S2 paradigm, namely product-[reviewer-review]. Specifically, we asked the subjects to imagine a scenario that they need purchase some goods for themselves or their families. While they were surfing on a social network such as Facebook, they noticed that there were some relevant messages posted by their friends or strangers who shared some products and provided their suggestions about them. Then after comprehensive consideration participants need determine whether they had a purchase intention towards the presented product. In this simulated social commerce shopping environment, we are better able to observe and obtain participants’ real reactions towards their friends and strangers.

In this paper, we primarily focus on using EEG technology to analyze the difference and change of ERP components under the friend and stranger condition. Also the behavior data is used as assistant support. Previous studies have revealed that fronto-central P2 component is sensitive to the fundamental cognitive and emotional processing [20, 21, 22], and posterior-parietal P3 component is related to high level cognitive activities such as decision making [23, 24, 25]. Hence in this experiment we speculated that P2 and P3, two ERP components sensitive to cognitive and emotional processes, would be recorded. The objective of our experiment is to collect evidence to support our hypothesis regarding consumers’ emotional and cognitive processing differences in handling different social ties. Further these findings can be used to assist social commerce websites and platform designers to arrange their platform layout and optimize platform function to be more reasonably, impressively and attractively to consumers.

## Method

### Participants

According to the ‘Chinese Online Shopping Market Research Report in 2013’ issued by China Internet Network Information Center (CNNIC), social commerce users in China have the following characteristics: (1) Age. Users aged 20–39 years old are the most active participants, and users aged 20 to 29 account for 56.4%; (2) Education. Users with master’s or a higher degree account for 41%, showing a high educational level. Therefore, in our experiments 23 right-handed participants (11 men and 12 women) in Beihang University with more than one-year usage experience of social commerce were chosen. Their ages ranged from 22 to 32 years old (mean age 26.6) and all were university-educated netizens. All the participants had normal or corrected-to-normal vision and no history of neurological or psychiatric illness. In the process of analysis, one female subject was excluded because she did not understand the experimental procedure very well and thus failed to provide effective data. Before starting, participants were informed of the experiment procedure and how the EEG data were being collected, and then they provided written informed consents. The Academic Review Board of Beihang University Behavior and Human Factors Engineering Lab reviewed and approved the research protocol.

### Materials

Before the start of experiment design, according to the ‘Chinese Online Shopping Market Research Report in 2014’ by CNNIC, clothing, digital product and commodity are the TOP 3 merchandises in online shopping. Combined with the age and education distribution of social commerce users and their preferences, we chose 20 products, including watch, digital camera, badminton racket, film and so on. Then another 37 candidates were asked to select 10 products that they had the most desire to buy in the near future. Results showed that watch, digital camera, tablet, laptop, film, delicious food, badminton racket, sport match ticket, travelling and art photo taking were the TOP 10 merchandises that participants had the greatest intention to buy, and thus they were chosen to comprise the product domain in our experiment. By communicating with selected participants, 10 females and 10 males who were their common and familiar peers were picked out as the friend domain from 20 females and 20 males who were their classmates in the same university. Additionally another 10 females and 10 males to whom participants were unfamiliar were chosen as the stranger domain from another 20 females and 20 males who were students from other universities. Front facial images with neutral expression of the selected 20 females and 20 males were collected as the reviewers’ portraits. The individuals whose photographs were displayed in the experiment had offered informed consent to use their photographs. Referring to the report in 2014 by CNNIC, the TOP 3 commonly used online shopping networks in China are Taobao, Tianmao and Jingdong. Therefore we collected online reviews about the 10 products from these three networks as the review domain, totaling 40 online reviews (4 reviews for each product). During the process of the experiment, the reviewer and review stimuli were arranged and displayed randomly. All the products and reviews were shown in the form of Chinese characters (black, Song font, size 20, less than 8 lengths). And all stimuli were processed digitally as 200*200 pixel, white background and the same luminance by Firework 8 (MacroMind Co., San Francisco, California, USA).

### Stimulus presentation and timing

In a dimly lit sound-attenuated room, participants were seated in a comfortable chair. Before start, experimental scenario and task were introduced to participants. There were 10 products they found by browsing a social network. Then they could know who shared it and what comment the person made. After knowing the review provider and reading his or her review, they should determine whether they had an intention to buy the product. Furthermore they were instructed to avoid body movement, especially eye blinking and movement and place their figures on the keyboard, the left index finger on the “F” key and the right index finger on the “J” key. Stimuli were shown from 1 meter at the center of a 20 inch monitor (1024*768 pixels, refresh rate 200 Hz) with a visual angle of 6°. Stimuli were controlled by E-prime 2.0 software (Psychology Software Tools Inc., Sharpsburg, Pennsylvania, USA) running on a 2.53 GHz Intel(R) Core(TM)2 computer.

**Fig 1:**
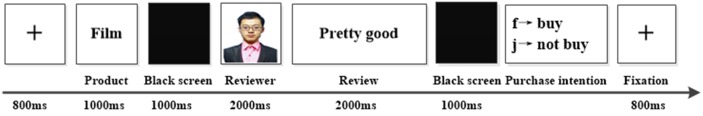
Flow diagram of the task.

80 S1-S2 (product-[reviewer-review], 10*2*4) pairs were used to investigate subjects’ purchase intention with the right and left index finger on the keyboard. The assignments of the response hands were counterbalanced across individuals, namely, half of participants used right index finger on the “F” key to express their “buy” intention and left index finger on the “J” key for “not buy” intention, while the other half used the opposite hands. Each trial started with a red fixation “+” lasting for 800ms to concentrate participants’ attention. Then the name of the product was flashed for 1000ms, followed by a 1000ms black screen. After that the reviewer’s portrait was displayed for 2000ms, followed by a review about the product for 2000ms. Another black screen for 1000ms was shown, and the final part was a “buy” or “not buy” question. Referring to the given product, reviewer and review, participants had to make their decisions without a time limitation, namely, the experiment would switch to the next new trial if and only if participants had made a decision and given their key press reaction. The experiment, lasting for a total of 12–15 minutes, included an exercise block of 4 trials and an experiment block of 80 trials. Fig 1 shows the flow diagram of one trial in the experiment. The individual in this manuscript has given written informed consent (as outlined in PLOS consent form) to publish these case details.

### EEG recording and analysis

EEG was recorded continuously along the scalp (online bandpass filter 0.3–100 Hz, sampling rate 500 Hz) using a 64-electrode HydroCel Geodesic Sensor Net 64 2.0, Net Amps 300 amplifier, and Net Station (Version 4.3.1) software. Behavior data was collected by E-prime 2.0 software. All channels were referred to the vertex sensor (Cz) during acquisition. The vertical electrooculogram (EOG) was recorded by two electrodes located above and below both eyes. The horizontal EOG was registered with lateral electrodes on the outer canthi of both eyes. Scalp impedance for each channel was maintained below 50 kΩ.

After acquisition, ERP components were analyzed offline by Net Station 4.3.1. Continuous EEG was filtered with 1Hz high-pass filter and 30Hz low-pass filter [26] and segmented by reviewer condition into 800ms stimulus-epochs from 200ms preceding the onset of reviewer stimulus and lasting for 600ms. After that artificial detection and correction were conducted to eliminate epochs that were contaminated by overlarge amplitude differences (±200 mV), vertical EOG (eye blinks; ±140uv) and horizontal EOG (±55uv). All marked bad channels were replaced with spherical splines interpolation based on all of the remaining channels in a given trial. Moreover, a segment was marked as bad if the number of bad channels were more than 10. All bad segments were rejected before averaging ERP data. The average data per channel was re-referenced to an average reference corrected for the polar average reference effect (PARE) [27]. And then the data was baseline-corrected for the mean voltage of 200 preceding the reviewer stimuli. The corrected data was used for the following within-participant repeated-measures analysis of variance (ANOVA).

## Results

### Behavioral data analysis

**Fig 2:**
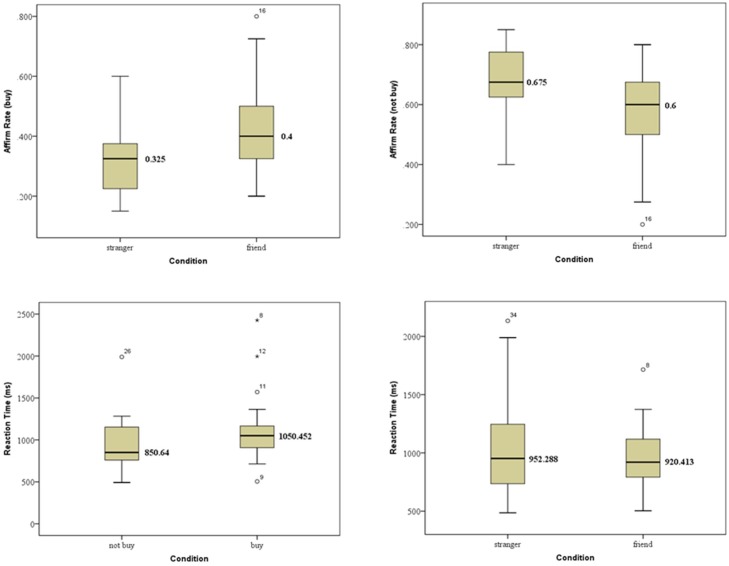
Behavior data distribution.

**Fig 3:**
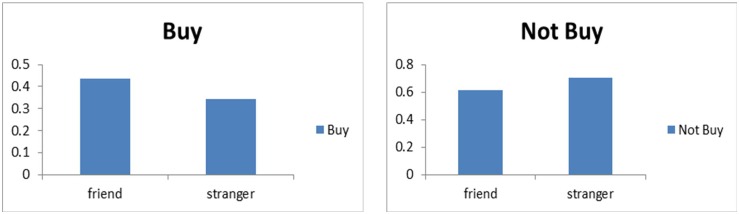
Purchase intention distribution.

All participants gave their responses, so there were 80 valid trials (10 products, for each one with 4 reviews, and for each review with 2 reviewers (a friend and a stranger)) for each subject. Using SPSS 19.0 (SPSS Inc., Chicago, Illinois, USA), statistical tests were conducted. The behavioral data distribution of two kinds of social ties (friend or stranger) and purchase decisions (buy or not buy) are shown in Fig 2. Affirmative decisions (buy or not buy) were different in two reviewer conditions. Specifically when the product was recommended by friends, there were higher “buy” decisions, while by strangers, the “not buy” decisions were more dominated. The specific distribution of purchase intention was shown in Fig 3: in “buy” condition, suggestions from friends contributed to more “buy” decisions, however, strangers’ suggestions made more participants decide not to buy the products. These findings were in accord with our intuition that due to more familiarity and trustworthiness to our friends, reviews from friends would be more prone to be accepted and contribute to making a purchase decision. While for reviews from strangers, consumers may be skeptical about their authenticities and hence hesitate to buy. Another valuable information we can obtain from behavioral data was the reaction time for purchase decision. From Fig 3, we found that reaction time for buy decision was longer than not buy decision. There was almost no difference between reaction time of purchase decisions referring to friends’ reviews and that referring to strangers’ reviews except that those from friends’ reviews were more concentrated.

The repeated-measures analysis of variance (ANOVA) in two social tie strengths (stranger and friend) and two purchase decisions (buy or not buy) indicated that the purchase decision difference was significant [F(1,21) = 20.903, P<0.001, *η*^2^ = 0.499], and social tie had a significant influence on purchase decision [F(1,21) = 25.310, P<0.001, *η*^2^ = 0.547]. Specifically in “buy” condition, friends’ effects were primary and statistically significant (friend (0.41±0.144) vs. stranger (0.33±0.132); [F(1,21) = 25.310, P<0.001, *η*^2^ = 0.547]); while in “not buy” condition, strangers’ effects were dominated and statistically significant (friend (0.58±0.144) vs. stranger (0.67±0.132);[F(1,21) = 25.310, P<0.001, *η*^2^ = 0.547]). Considering reaction time, it was shorter in “not buy” condition than in “buy” one [not buy (943±336) vs. buy (1125±429)], and the discrepancy was statistically significant [F(1,21) = 5.14, P = 0.034, *η*^2^ = 0.197], accordant with our intuition that more consideration is taken to make a purchase decision. Moreover the reaction time of purchase decision was shorter in friend condition than in stranger one [friend (967±297) vs. stranger (1047±419)], but it was not statistically significant [F(1,21) = 1.585, P = 0.222, *η*^2^ = 0.07]. One possible reason is that when people see their friends or strangers, time to detect, evaluate and recognize may be different, but when they are ready to make a purchase decision, they are more likely to rely on comprehensive consideration.

### ERPs

**Fig 4:**
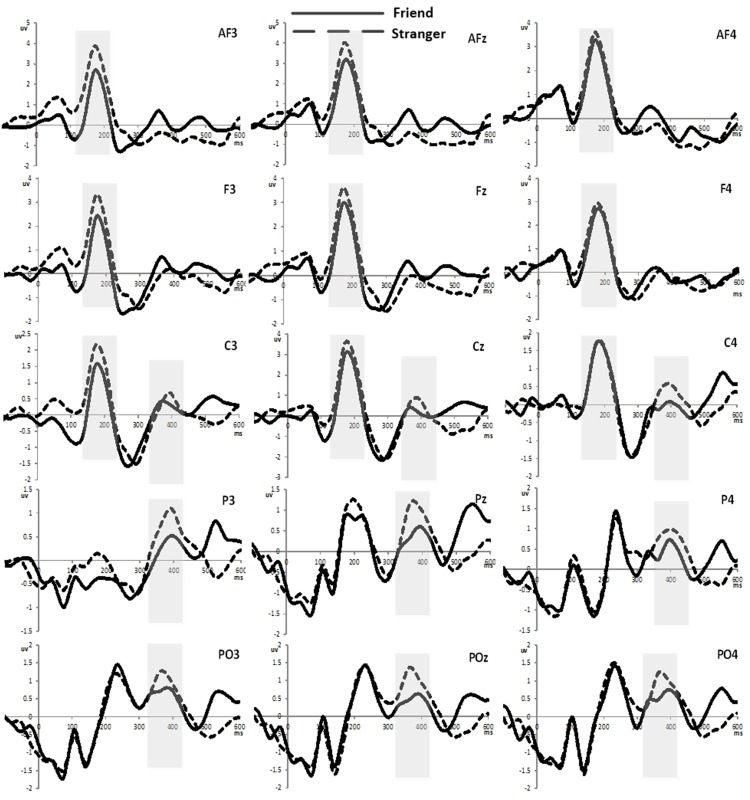
Grand averaged ERP at fifteen selected electrodes in two conditions.

**Fig 5:**
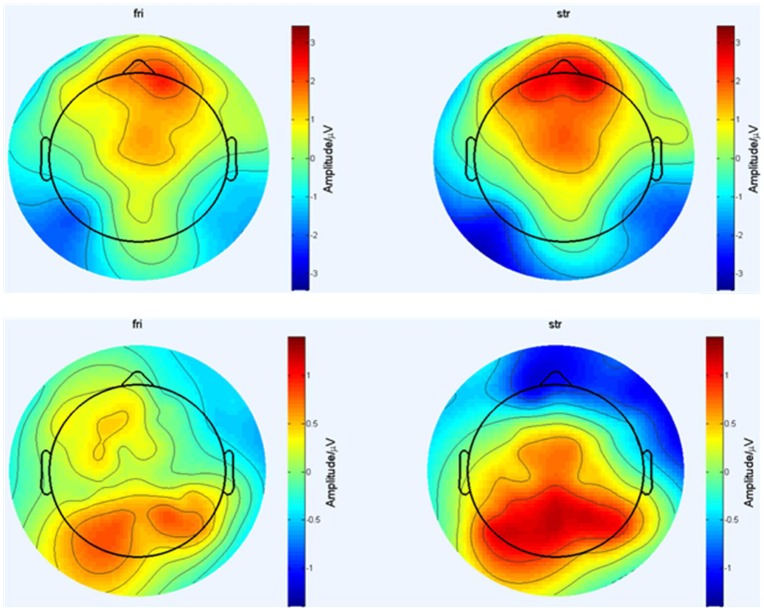
Topographic maps of the maximal amplitudes of the P2 (150-250ms) and P3 (350-400ms).

**Fig 6:**
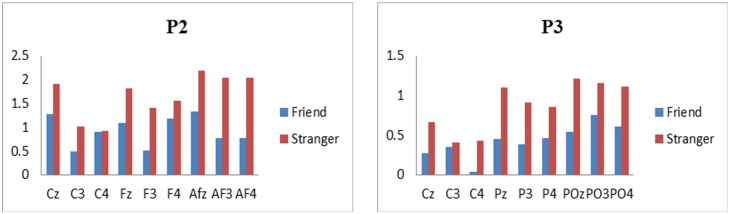
Mean amplitudes of ERP recorded at each electrode.

Fig 4 shows grand-average ERP waveforms in two conditions in the frontal region (AF3, AFz, AF4, F3, Fz, F4), central region (C3, Cz, C4) and posterior-parietal region (P3, Pz, P4, PO3, POz, PO4). Fig 5 shows the brain topographic maps based on the average across multiple time points (150-250ms and 300-450ms). In a topographic map, different colors indicate different potential levels evoked in the brain, which can represent the magnitude of activation in the brain area. For positive potentials, more red color manifests higher active level, followed by yellow and green. For negative potentials, more blue color indicates more active in this area. Regarding time window 150-250ms, frontal and central areas show more red color than other areas, which means during this time a positive component was evoked in these ranges of the brain areas. For time 300-450ms, central and parietal areas manifest more red color than other areas, indicating in these areas of the brain a positive component was elicited during this time period. Many studies related to facial recognition [28,29], emotion processing [20,21] and task-relevant events [12,13] which are involved in both cognitive and emotional processing have reported the amplitude and latency of some commonly elicited ERP components such as N1, P2, P3, late positive potential (LPP). As supposed, we detected that P2 and P3 components were elicited successfully in our results. Previous literatures have generally reported that P2 are recorded from frontal, central and parietal scalp areas [20,26] and P3 are topographically maximal in the centero-parietal electrode sites [30,31]. For statistical analysis, we focused on 9 fronto-central electrodes AF3, AFz, AF4, F3, Fz, F4, C3, Cz, C4 where P2 is recorded generally and 9 central and posterior-parietal electrode sites C3, Cz, C4, P3, Pz, P4, PO3, POz, PO4 which are defining electrode sites for P3 components. Average amplitudes over these electrodes in each region were used in the following analysis and the time window 150-250ms for P2 and 350-400ms for P3 were determined referring to previous research and visual inspection in the present experiment. By using mean P2 amplitudes (150-250ms) and mean P3 amplitudes (350-400ms) in two conditions, a within-participants repeated-measure ANOVA with 2 (tie strengths) *9 (scalp channels) was operated. The Greenhouse-Geisser correction was applied. Following the onset of the reviewer block, the positive P2 and P3 components were recorded for each condition. Within each given time window, the mean amplitudes of ERP in each channel for the two conditions are displayed in Fig 6.

### P2 (150-250ms)

As shown in Fig 4, the positive components P2 were obviously observed in each fronto-central channel for the two conditions. The analysis of P2 mean amplitudes showed a significant main effect of social tie [F(1,21) = 12.981, P = 0.002, *η*^2^ = 0.382]. Also the main effect of electrode sites was significant[F(8,14) = 4.817, P = 0.005, *η*^2^ = 0.734]. However, the electrode sites did not interact with tie strength [F(8,14) = 1.382, P = 0.285, *η*^2^ = 0.441]. In addition, from the descriptive statistics in Fig 6 the average amplitudes of P2 (150-250ms) in stranger condition were larger than those in friend condition over all nine channels, reaching its maximum at the midline AFz, Fz, Cz. And the difference of mean amplitudes between two conditions was significant ([F(1,21) = 12.981, P = 0.002, *η*^2^ = 0.382]; friend (0.998±1.51) vs. stranger (1.645±1.39)). Additionally, the analysis of P2 latency did not yield significant main or interaction effects.

### P3 (350-400ms)

As shown in Fig 4, the positive P3 components were obviously discerned from each central and posterior-parietal channel for the two conditions. The repeated measures ANOVA of mean amplitudes of P3 components showed that there was a significant main effect of tie strength [F(1,21) = 5.122, P = 0.034, *η*^2^ = 0.196]. But there did not yield significant main effect of electrode sites [F(8,14) = 2.052, P = 0.114, *η*^2^ = 0.540] and their interactive effect [F(8,14) = 1.275, P = 0.33, *η*^2^ = 0.421]. Additionally, according to descriptive statistics in Fig 6, the mean P3 amplitudes in stranger condition were larger than those in friend condition over all nine channels too, and reached its peak at the midline Cz, Pz, POz. Also this difference of average P3 amplitudes for two conditions was significant ([F(1,21) = 5.122, P = 0.034, *η*^2^ = 0.196]; friend (0.422±0.98) vs. stranger (0.871±0.93)). The analysis of P3 latencies indicated a salient difference for two conditions, and longer latencies were recorded in stranger condition than that in friend condition ([F(1,21) = 19.455, P<0.001, *η*^2^ = 0.481]; friend (366.045±22.78) vs. stranger (383.783±20.09)). No other effects were shown.

## Discussion

Generally according to ERP measure, the amplitudes of ERP components demonstrate the degree or intensity of the engagement of cognitive processes, and latencies are supposed to manifest the time course of processing stage [20]. Sensory component P2 seemingly involves in the fundamental neural and emotional processing activities automatically. It is indicative to evaluate the emotionality and importance of stimuli [20], and to reflect input processing-related attention [32]. In addition, positive potential P3 provides a variety of information about the neural activities of elementary cognitive operations, especially the information processing associated with memory [23,24] and attention [25] mechanisms. Its amplitude is an indicator of the attention resources modulation [33], and its peak latency is a reflection of stimulus classification time [34].

### P2: Affection processing and attention resource allocation

In fact, P2 is sensitive to the emotional stimuli [22] and primarily evaluates the affective content of stimuli [20]. A considerable number of articles have reported the emotional evaluation processes reflected by P2 amplitude modulation. According to Zajonc [35], the early assessment of affective connotation is processed automatically and independent of conscious inferences. P2 amplitude modulation can indicate the global affective evaluation [21], and its magnitude can reflect a general evaluation of emotional significance [20]. This line of studies discovered that compared with feelings aroused by positive stimuli, augmented P2 amplitudes were elicited in response to negative stimuli. In our study, we also had this “negative bias”. Specifically seeing friends’ portraits may lead participants to feel some positive emotions such as comfort, happiness and trust, while looking at strangers’ images may prompt them to feel some negative affections such as worry, repulsion and distrust, resulting in higher P2 amplitudes. Thus augmented P2 components under stranger condition implied that strangers’ portraits triggered more negative affections.

Furthermore, P2 over fronto-central scalp areas is considered as an attention-related component, which can indicate automatic mobilization of attention resources [21, 32]. Because 200ms is an early time stage and P2 is an indicator at the boundary of unconsciousness and consciousness, the attention bias can occur automatically [21]. Additionally, some findings have elucidated that more attention resources are mobilized to emotional than to neutral stimuli [28], specifically to negative stimuli than to positive ones [21, 36]. In our study, more positive P2 amplitudes elicited by stranger stimuli suggested that the cortical neural response to stranger stimuli was activated more due to our self-protection and alert awareness. Therefore more attention resources were garnered when facing strangers’ images in order to facilitate subsequent psychological and behavior reactions. However, due to possibly automatic emotional and cognitive processing indicated by P2, the latency difference between friend condition and stranger condition was not apparent.

### P3: Motivation-related attention resource mobilization

Generally, P3 arises during later, high level, cognitive stages when cognitive operations related to selective attention [23] and attention resource allocation are engaged. Its amplitudes are proportional to the amount of attentional resources involved in processing given stimuli [37], and its latency can reflects the time of stimulus classification, which is matching with the time required to detect and assess stimuli [34]. Response to a stimulus, P3 marks the preferential allocation of attention resources to potentially relevant events. This motivated attention is evoked by stimuli that can trigger motivational processes such as approach or avoidance [38]. In our study, stranger stimuli were noticed selectively and occupied more attentional resources, resulting in a longer latency. One possible explanation is that compared with friend stimuli, triggering feelings like happiness, comfort and trust, stranger stimuli may be less positive due to evoking affections like alertness, uneasiness and distrust. Therefore a close scrutiny may continue long into the period of strangers’ portraits exposure. The sustained call on attention resources is presumably related to the complex array of behavioral, emotional and peripheral physiological response manipulated by the brain’s appetitive and defensive motive systems [38].

Our finding is inconsistent with some previous studies [28, 29] due to the different experiment tasks. In our study, the perception of friends and strangers is implicit and is served to the shopping task. Participants’ missions are not just to recognize that the person presented is their friend or stranger. They may more focus on the experiment condition that they need to buy something and this person provided a product and relevant review. However, in the typical facial recognition experiments, participants are required to react to certain stimuli (e.g. response to friends’ portraits) [28] or to state their judgement on the person presented (e.g. the person is your: 1.friend 2.stranger) [29], which were explicit to identify friends. Additionally the reverse results between Bai’s experiment [4] and our study might be caused by different way to manipulate S2 stimuli. In their study, reviewer and review stimulus was arranged as an integral, thus more pronounced amplitudes and larger latency of P3 components in review condition from friends cannot be simply explained as the difference of information processing between friends and strangers because it was a compound effect of the review and reviewer. In our study, however, we inspected the independent effect of friend or stranger excluding the intervention of other stimuli.

## Conclusion

Opening the black box of the brain has the potential to enhance our understanding about how friends and strangers influence consumers during their purchase processes, as this article attests. To deploy our work, a S1-S2 paradigm (product-[reviewer-review]) was designed, where participants provided their purchase intention choice by pressing corresponding keys after comprehensively considering all given information (product, reviewer and review). The perception, evaluation and processing of friend and stranger stimuli modulated the P2 and P3 components. In conclude, the augmented P2 and P3 components under stranger condition indicated that there was obviously different neural processing engagement in the brain to handle friend and stranger stimuli. P2, as an early ERP component, can reflect some cognitive processes, such as the evaluation of affective connotation and attention resources allocation. The more positive P2 component evoked by stranger stimuli demonstrated that the emotion evaluation related to the friend and stranger stimuli occurred during consumer purchasing process, and more attention resources were allocated to strangers to detect and facilitate subsequent physiological and behavioral operations. In additionally, previous studies have elucidated that P3 is provoked by a distributed network of the brain to process activities associated with attention and memory operations. Conspicuous P3 amplitudes under stranger condition demonstrated that evaluating, categorizing and processing stranger stimuli manipulated more attention resources, resulting in a close and continued scrutiny into stranger stimuli and indicating a larger latent period. In conclusion, the neurocognitive response course to friend and stranger stimuli during consumer purchasing are mainly related to two stages: affection evaluation (P2) and attention modulation (P2 and P3). Therefore, our hypothesis has been supported that processing social tie stimuli (friends and strangers) would engage both cognitive and emotional processes in the brain (supported by successfully evoking P2 and P3 components), and the magnitude of activation in both cognitive and emotional processing might explain the subsequent consumer decisions to accept or reject a product (supported by magnitude differences of P2 and P3 components under friend and stranger conditions and behavior data of purchase decision).

The present ERP results yield new insights into interpreting neural fundament of previous finding that friends exert more influence than strangers over consumers’ decision making. Our study suggested during this process emotion evaluation plays a key role. Compared to friends who are prone to trigger more positive emotions such as comfort, happiness and trust, strangers are more likely to evoke negative bias affections such as uneasiness, repulsion and distrust. It is this negative-bias emotion that makes suggestions from strangers less impressive, trustworthy and reliable and weakens their influential power. The attention resource allocation also impact consumers’ decision. Therefore in social commerce context where users communicate mostly with their friends, consumers will enjoy a more comfort, relaxing and secure shopping experience, which will contribute to stronger purchase intention. This is one of outstanding competitive advantages of social commerce which makes it more promising. Meanwhile our findings have important management suggestions for social commerce managers. For example, social commerce platforms could leverage on social tie data to help consumers filter the usefulness of information and identify and push their interesting information. By using the tie strength effect and filter mechanism, consumers can find their interesting information more quickly, and also companies can find their target consumers more easily.

Although the behavior and EEG data have provided convictive evidence to support our hypothesis that friends and strangers have different influence during the process of consumer purchase decision, there are still many relevant studies can be done further to complement and improve our study. For example, in social networks people almost know all their fellows, merely with different intimacy, familiarity and trust. However, in the present study we only grouped fellows as familiar ones (friends) and unfamiliar ones (strangers). Future work can categorize friends into more detailed familiarity ranks (little familiar, more familiar, very familiar) to explore the influence of precisely divided social ties on purchase decision. Additionally noting that some research have declaimed friend dominated effect was weakened in certain conditions such as low-risk products [9] and utilitarian products [11]. Moreover, the valence of review is an indispensable factor to influence the social tie effect during consumer decision making. Therefore further investigations should consider more specific factors to reveal the neural underpinning of social tie effect and to provide more guidance for managing and running social commerce.
